# Achieving blood pressure control targets in hypertensive patients of rural China – a pilot randomized trial

**DOI:** 10.1186/s13063-020-04368-1

**Published:** 2020-06-11

**Authors:** Xiao Huang, Lishun Liu, Yun Song, Lan Gao, Min Zhao, Huihui Bao, Xianhui Qin, Yanqing Wu, Qinghua Wu, Chonglei Bi, Aiping Yue, Chongqian Fang, Hai Ma, Yimin Cui, Genfu Tang, Ping Li, Yan Zhang, Jianping Li, Binyan Wang, Xiping Xu, Hong Wang, Gianfranco Parati, J. David Spence, Xiaobin Wang, Yong Huo, Guangliang Chen, Xiaoshu Cheng, for the investigators of intensive BP control in China

**Affiliations:** 1 Department of Cardiology, The Second Affiliated Hospital of Nanchang University, Nanchang, China; 2 Advanced Innovation Center for Food Nutrition and Human Health, College of Food Science and Nutritional Engineering, China Agricultural University, Beijing, China; 3 National Clinical Research Study Center for Kidney Disease, State Key Laboratory of Organ Failure Research, Renal Division, Nanfang Hospital, Southern Medical University, Guangzhou, China; 4 Department of Cardiology, Peking University First Hospital, Beijing, China; 5 Department of Neurology, Guangdong Provincial Hospital of Chinese Medicine, Guangzhou, China; 6 Prevention and Control Office of Chronic Disease in Rongcheng, Rongcheng, Shangdong China; 7 Disease Control and Prevention Center, Rongcheng, Shandong China; 8 People’s Hospital of Rongcheng, Rongcheng, Shandong China; 9 Health and Family Planning Commission, Rongcheng, Shandong China; 10 Department of Pharmacy, Peking University First Hospital, Beijing, China; 11 Health Management College, Anhui Medical University, Hefei, China; 12 Institute of Biomedicine, Anhui Medical University, Hefei, China; 13 Centers for Metabolic Disease Research, Temple University School of Medicine, Philadelphia, PA USA; 14 Department of Cardiovascular, Neural and Metabolic Sciences, S. Luca Hospital, Milan, Italy; 15 Stroke Prevention and Atherosclerosis Research Centre, Robarts Research Institute, Western University, London, ON Canada; 16 Department of Population, Family and Reproductive Health, Johns Hopkins University Bloomberg School of Public Health, Baltimore, MA USA; 17 College of Integrated Traditional Chinese and Western Medicine, Anhui University of Chinese Medicine, Hefei, China

**Keywords:** Intensive blood pressure control, Feasibility, Home blood pressure measurement, Rural China

## Abstract

**Background:**

This study aimed to test the feasibility and titration methods used to achieve specific blood pressure (BP) control targets in hypertensive patients of rural China.

**Methods:**

A randomized, controlled, open-label trial was conducted in Rongcheng, China. We enrolled 105 hypertensive participants aged over 60 years, and who had no history of stroke or cardiovascular disease. The patients were randomly assigned to one of three systolic-BP target groups: standard: 140 to < 150 mmHg; moderately intensive: 130 to < 140 mmHg; and intensive: < 130 mmHg. The patients were followed for 6 months.

**Discussion:**

The optimal target for systolic blood pressure (SBP) lowering is still uncertain worldwide and such information is critically needed, especially in China. However, in China the rates of awareness, treatment and control are only 46.9%, 40.7%, and 15.3%, respectively. It is challenging to achieve BP control in the real world and it is very important to develop population-specific BP-control protocols that fully consider the population’s characteristics, such as age, sex, socio-economic status, compliance with medication, education level, and lifestyle. This randomized trial showed the feasibility and safety of the titration protocol to achieve desirable SBP targets (< 150, < 140, and < 130 mmHg) in a sample of rural, Chinese hypertensive patients. The three BP target groups had similar baseline characteristics. After 6 months of treatment, the mean SBP measured at an office visit was 137.2 mmHg, 131.1 mmHg, and 124.2 mmHg, respectively, in the three groups. Home BP and central aortic BP measurements were also obtained. At 6 months, home BP measurements (2 h after drug administration) showed a mean SBP of 130.9 mmHg in the standard group, 124.9 mmHg in the moderately intensive group, and 119.7 mmHg in the intensive group. No serious adverse events were recorded over the 6-month study period. Rates of adverse events, including dry cough, palpitations, and arthralgia, were low and showed no significant differences between the three groups. This trial provided real-world experience and laid the foundation for a future, large-scale, BP target study.

**Trial registration:**

Feasibility Study of the Intensive Systolic Blood Pressure Control; ClinicalTrials.gov, ID: NCT02817503. Registered retrospectively on 29 June 2016.

## Background

Hypertension is highly prevalent in the world, particularly in China [1–3] and is a leading modifiable cause of end-organ damage, including stroke, cardiovascular disease, and chronic kidney disease [4–8]. Clearly, achieving optimal blood pressure (BP) control is critically important to prevent hypertension-induced end-organ diseases. Yet, questions and challenges persist in attaining this goal. First, there is still no consensus as to what is the optimal BP target in the general population for the primary prevention of stroke and cardiovascular disease for people over 60 years of age. The Joint National Committee on Prevention, Detection, Evaluation, and Treatment of High Blood Pressure (JNC 8) [9] recommended initiating pharmacological treatment to lower BP to achieve a systolic blood pressure (SBP) target of < 150 mmHg and a diastolic blood pressure (DBP) target of < 90 mmHg for the general population aged ≥ 60 years, and a SBP target of < 140 mmHg and a DBP target of < 90 mmHg for people aged < 60 years of age. The results of the Systolic Blood Pressure Intervention Trial (SPRINT) [10] in which the investigators targeted a SBP of < 120 mmHg, as compared to < 140 mmHg, renewed interest on more intensive antihypertensive therapies directed towards a lower BP target among patients at high risk for cardiovascular events but without diabetes. The SPRINT trial showed lower rates of fatal and nonfatal major cardiovascular events and death in the lower BP target group, which subsequently led to a revision of the definition of hypertension in the American Hypertension Association Guidelines in 2017 [11]. Opponents argued that over-reduction of BP could lead to adverse events (AEs) such as severe hypotension and end organ hypo-perfusion or ischemia in some patients, particularly those with a pulse pressure > 60 mmHg and a DBP < 60 mmHg [12]. Thus, the optimal target for BP control remains a controversial topic and requires further evidence to weigh the benefits and risks.

Second, it is well-observed that hypertensive patients are heterogeneous by age, sex, race, ethnicity, risk factors, and co-morbidities. As such, when setting BP-control targets, it is important to carefully consider these factors, with the understanding that one size does not fit all. For example, there are several unique characteristics that are specific to hypertensive populations in China, including folate insufficiency, a high rate of the *MTHFR C677T* genotype mutation and a high rate (74.45%) of hyperhomocysteinemia (HHcy) [13–15].

Third, in real-world practice, achieving BP control and attaining the optimal target in a high-risk population, such as the rural Chinese, remain a challenge. Recent surveys have shown that the rates of awareness, treatment, and control are only 46.9%, 40.7%, and 15.3%, respectively, in China [2, 3]. Thus, there is an urgent need for evidence-based guidelines to inform clinical and public health practice and policy in rural China.

As a prelude to a large trial of BP targets, this pilot randomized trial aimed to test the feasibility of a BP-control protocol designed to effectively and safely manage hypertensive patients and achieve prespecified SBP targets in hypertensive patients of rural China. The hypotheses is whether this has the feasibility of achieving mean BP levels in each of the treatment groups in this present trial. In order to explore the various possibilities and the low rates of awareness, treatment, and control of BP in the rural Chinese population, a three-group design (i.e., 140 to < 150 mmHg, 130 to < 140 mmHg, and < 130 mmHg) was made in this pilot study. Furthermore, different modalities for obtaining BP measurements (routine office visits, home blood pressure measurement (HBPM), and central aortic systolic pressure (CASP)) were also included as part of the trial.

## Methods

### Study design and oversight

The study was a randomized, controlled, open-label trial conducted in Rongcheng, Shandong, China. The trial consisted of three stages: (1) screening, (2) recruitment and randomization to specific BP targets, and (3) antihypertensive treatment titrated to achieve the assigned BP target. The study was approved by the Ethics Committee of the Second Affiliated Hospital of Nanchang University, China and is registered in the clinical trials website (ClinicalTrials.gov, ID: NCT02817503). All participants provided written, informed consent.

### Study population

#### Inclusion criteria

1. Hypertensive patients aged 60 years or older
2. Current SBP ≤ 150 mmHg but < 180 mmHg (within the previous 2 weeks) and not regularly treated with antihypertensive drugs
3. If currently regularly treated with antihypertensive drugs (at least 10 days on antihypertensive drugs within the previous 2 weeks), BP must meet the following criteria:
  SBP ≥ 140 mmHg but < 170 mmHg, if regularly (no less than 10 days) taking one type of antihypertensive medication within the previous 2 weeks
  SBP ≥ 130 mmHg but < 160 mmHg, if regularly (no less than 10 days) taking two types of antihypertensive medication within the previous 2 weeks
  SBP ≥ 130 mmHg but < 150 mmHg, if regularly (no less than 10 days) taking three types of antihypertensive medication within the previous 2 weeks
  For patients who were taking a fixed-dose combination (FDC), this treatment was considered as two types of drugs if the dose of each component of the FDC was equal to or higher than the routine therapeutic dose
4. Serum homocysteine (Hcy) ≥ 10 μmol/L, or the patient is taking enalapril-folic acid
5. Subject has read, agreed to, and signed a written, informed consent form

#### Exclusion criteria

1. History of physician-diagnosed stroke, myocardial infarction, heart failure, revascularization, or malignancy
2. History of physician-diagnosed secondary hypertension
3. Undergoing regular renal dialysis or has been diagnosed with end-stage kidney disease
4. Congenital or acquired organic heart disease
5. Severe somatic disease preventing the participant from completing the trial, or the patient is incapable of participating, as judged by the investigators
6. Contraindications or intolerance to angiotensin-converting-enzyme inhibitors (ACEIs) (including enalapril-folic acid) or, a history of severe adverse effects to ACEIs
7. Abnormal laboratory test results and/or clinical manifestations rendering the patient unsuitable to participate as judged by the investigators

### Randomization and interventions

During the screening stage, each participant completed a physical examination and questionnaire interview on lifestyle and history of disease and medication use. Laboratory tests included fasting lipid profile and plasma Hcy. Eligible participants were randomized, in a 1:1:1 ratio, to a SBP target of 140–150 mmHg (the standard-treatment group (Group A)), 130–140 mmHg (the moderately-intensive treatment group (Group B)) or < 130 mmHg (the intensive-treatment group (Group C)) with a fixed block size of 9. Study personnel were aware of the study-group assignments, but participants were not.

After the participants had undergone randomization, their baseline antihypertensive regimens were adjusted on the basis of the study-group assignment. The treatment algorithms were similar to those used in the SPRINT trial [10]. These algorithms and our formulary are listed in the supplemental material (Supplemental Table 1). All major classes of antihypertensive agents were included in the formulary and were provided at no cost to the participants. For all participants, the initial therapy was a daily oral dose of one tablet of enalapril-folic acid (containing 10 mg enalapril and 0.8 mg folic acid). Other drugs, including calcium-channel blockers (CCBs) (amlodipine preferred), diuretics (hydrochlorothiazide preferred), and β-blockers, were allowed, in order to achieve the SBP target. For those who could not tolerate enalapril-folic acid well, other types of antihypertensive agents could be used as alternative choices. If the target BP level was not achieved during the titration or follow-up periods, adjustment of drug type and dosage was carried out according to the protocol.

Participants were seen weekly for the first month, every 2 weeks for the next 2 months, and once a month thereafter for a total of 6 months, totaling 11 follow-up visits. For participants in Groups A and B, medications were adjusted to a target SBP of 145–149 mmHg and 135–139 mmHg, respectively. For participants in Group C, medications were adjusted to a target SBP of < 130 mmHg. The dose was reduced if the SBP was under the target on two consecutive visits. Dose adjustment was based on the mean of three BP measurements at an office visit. Self-monitored HBPM was recorded by using an electronic sphygmomanometer (Kingyield, Shenzhen, China). Additionally, CASP measurement was also conducted at office visits by using a CASP monitoring device (A-pulse CASPro, Jianzi, Singapore). Lifestyle modifications, like sodium restriction and smoking cessation, were encouraged as part of the management strategy for all study participants. The participants’ retention and adherence to treatment were also monitored at each follow-up visit.

### Outcomes and study measurements

The primary outcome was achieved mean BP levels in each of the treatment groups. The secondary outcome was the difference between the carotid-femoral pulse-wave velocity (cf-PWV), 3D carotid artery ultrasound, and Ankle-brachial Index (ABI) of each treatment group. BP measurements at an office visit were with the patient seated and having rested quietly for 10 min; the measurements were made with the use of an electronic sphygmomanometer (Kingyield, Shenzhen, China). Self-monitored HBPM was recorded by using an electronic sphygmomanometer (Kingyield, Shenzhen, China). Participants were trained on the use of the electronic sphygmomanometer for HBPM according to the protocol. Before formally recording any BP values, the patients underwent 10 days of continuous training conducted by the investigators to ensure that each participant mastered the method. The participants were requested to continuously measure BP at three time points per day (in the morning after urination and before breakfast and medication, 2 h after taking antihypertensive medication, and in the evening) for weeks 12, 16, 20, and 24, and to record the values on a HBPM self-test registration form. All data were collected 7 days before the office visit. The main objective of the HBPM protocol was to assess the reproducibility and reliability of 7-day self-monitoring prior to an office visit day. CASP was also measured at the office visits at weeks 6, 12, and 24 by using a CASP monitor device (A-pulse CASPro, Jianzi, Singapore). Epidemiological, clinical, and laboratory data were obtained at baseline. Data on cf-PWV, 3D carotid artery ultrasound, and ABI were all obtained at baseline and at the 6-month visit. Medical records and electrocardiograms were obtained for documentation of events. Serious adverse events (SAEs) were defined as events that were fatal or life-threatening, that resulted in clinically significant or persistent disability, that required or prolonged a hospitalization, or that were judged by the investigator to represent a clinically significant hazard or to cause harm to the participant, that might require medical or surgical intervention to prevent one of the other events listed above. Any condition on a short list of monitored conditions would be reported as an AE if it was evaluated in a hospital emergency department: hypotension, syncope, injurious falls, electrolyte abnormalities, and bradycardia.

### Statistical analysis

According to previous large-scale randomized controlled trial (RCT) studies, the rate of achieved mean BP levels in the target window is around 60%, assuming that the rate of achieved mean BP levels in this feasibility present study is 70–80% and the significance level of bilateral test *α* = 0.05. With an enrollment target of 30 participants of each group, we estimated that the trial would have 80% power to detect the difference between groups. We anticipated a loss to follow-up, so 35 participants were included in each group. Continuous variables were presented as mean ± standard deviation (SD) and categorical variables as frequency (%). The baseline population characteristics of the three BP groups were compared using the Kruskal-Wallis test or *χ*^2^ tests. Similarly, the incidence of AEs that were likely caused by the drug or intensive BP control was compared among the three BP target groups. SBP and DBP were compared between 0- and 6-month points by paired *t* tests within each treatment group. Change in SBP and DBP (6-month BP minus baseline BP) was compared among the three treatment groups. Epidata 3.1 was used to build the database; double-entry mode and error checking were adopted. Data were analyzed using Empower (R) (www.empowerstats.com; X&Y Solutions, Inc., Boston, MA, USA).

## Results

### Characteristics of the participants

**Fig. 1:**
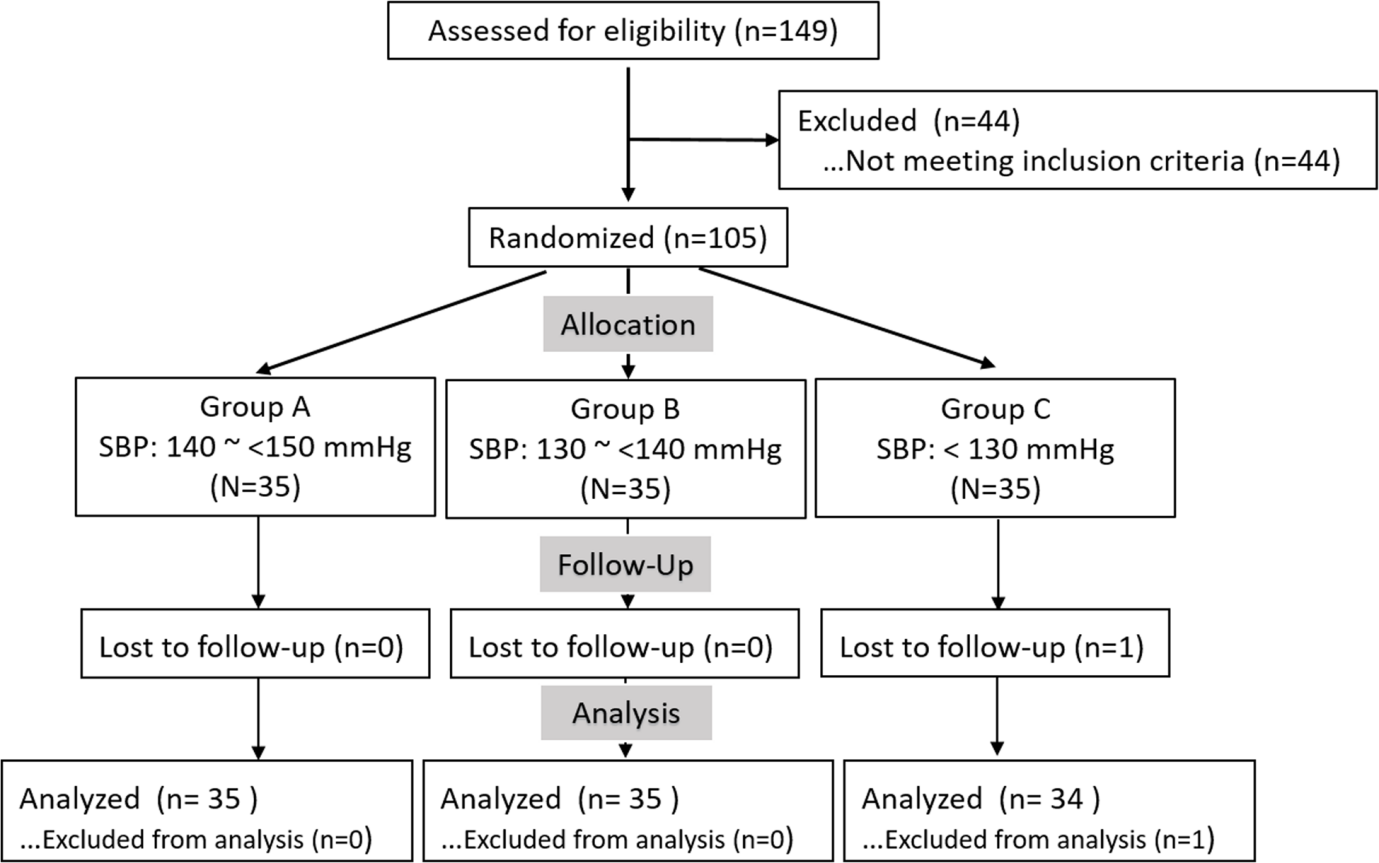
Design and flow chart of the feasibility study

One hundred and five 105 participants were enrolled between December 2015 and January 2016. Participants were randomized into three groups with different BP targets (Fig. 1). Baseline characteristics are shown in Table 1. The mean age of the population was 68.4 ± 5.5 years. Male participants constituted 31.4% of the participants, and 20% were former or current smokers; 15.2% of the participants had a history of diabetes. The percentage of SBP > 150 mmHg, > 140 but ≤ 150 mmHg; and > 130 but ≤ 140 mmHg at enrollment was 41.0%, 45.7%, and 13.3%, respectively. Aspirin and statins use was 5.7% and 20.0%, respectively. There were no significant differences in baseline characteristics among the three groups (*P* > 0.05).

**Table 1:** Characteristics of the participants at baseline according to BP-control group

	Total	A Standard (140 ~ < 150 mmHg)	B Moderate (130 ~ < 140 mmHg)	C Intensive (< 130 mmHg)	***P*** value*
**Number**	105	35	35	35	
**Age (years)**					
Overall	68.4 ± 5.5	67.4 ± 4.9	68.2 ± 5.8	69.5 ± 5.8	0.259
≥ 75 years of age	17 (16.2%)	3 (8.6%)	6 (17%)	8 (22.9%)	
**Baseline blood pressure (mmHg)**					
Systolic	149.6 ± 11.6	146.7 ± 8.6	147.2 ± 7.0	150.6 ± 8.5	0.098
Diastolic	89.2 ± 9.7	87.3 ± 9.1	88.5 ± 9.5	89.7 ± 10.6	0.581
**BMI**	26.5 ± 3.4	25.8 ± 3.3	26.9 ± 3.8	26.8 ± 3.1	0.346
**Sex**					1
Male	33 (31.4%)	11 (31.4%)	11 (31.4%)	11 (31.4%)	
Female	72 (68.6%)	24 (68.6%)	24 (68.6%)	24 (68.6%)	
**Smoking status**					0.446
Never	84 (80.0%)	27 (77.1%)	27 (77.1%)	30 (85.7%)	
Former	12 (11.4%)	6 (17.1%)	3 (8.6%)	3 (8.6%)	
Current	9 (8.6%)	2 (5.7%)	5 (14.3%)	2 (5.7%)	
**Diabetes history**	16 (15.2%)	6 (17.1%)	3 (8.6%)	7 (20.0%)	
**Distribution of systolic blood pressure (number %)**					0.143
> 150 mmHg	43 (41.0%)	11 (31.4%)	12 (34.3%)	20 (57.1%)	
> 140 ≤ 150 mmHg	48 (45.7%)	17 (48.6%)	19 (54.3%)	12 (34.3%)	
> 130 ≤ 140 mmHg	14 (13.3%)	7 (20.0%)	4 (11.4%)	3 (8.6%)	
**Antihypertensive drug use**	1.4	1.4	1.4	1.5	0.923
**Other medication usage**					
Statins	6 (5.7%)	1 (2.9%)	3 (8.6%)	2 (5.7%)	
Aspirin	21 (20.0%)	6 (17.1%)	7 (20%)	8 (22.9%)	
**Laboratory results**					
Glucose (mmol/L)	6.5 ± 1.7	6.7 ± 1.8	6.4 ± 2.0	6.3 ± 1.3	0.559
Cholesterol (mmol/L)	5.5 ± 1.5	5.1 ± 1.8	5.6 ± 1.0	5.8 ± 1.5	0.103
Triglycerides (mmol/L)	1.8 ± 1.5	2.0 ± 2.2	1.7 ± 1.1	1.8 ± 0.9	0.575
LDL (mmol/L)	4.1 ± 0.9	3.9 ± 0.9	4.0 ± 0.9	4.3 ± 0.9	0.307
HDL (mmol/L)	1.5 ± 0.3	1.5 ± 0.3	1.5 ± 0.3	1.5 ± 0.3	0.913
Homocysteine (μmol/L)	10.4 ± 2.6	10.6 ± 3.2	9.9 ± 1.9	10.5 ± 2.7	0.496
***MTHHR C677T***					0.679
CC	24 (22.9%)	7 (20.0%)	7 (20.0%)	10 (28.6%)	
CT	52 (49.5%)	20 (57.1%)	18 (51.4%)	14 (40.0%)	
TT	29 (27.6%)	8 (22.9%)	10 (28.6%)	11 (31.4%)	

Data are mean (SD) or number (%)

*ACE* angiotensin-converting enzyme, *ARB* angiotensin-II-receptor blocker, *BMI* body mass index, *BP* blood pressure, *MTHFR* methylenetetrahydrofolate reductase, *LDL* low-density lipoprotein, *HDL* high-density lipoprotein

*Difference between groups *p* < 0·0001

### BP titration and antihypertensive drug use

**Fig. 2:**
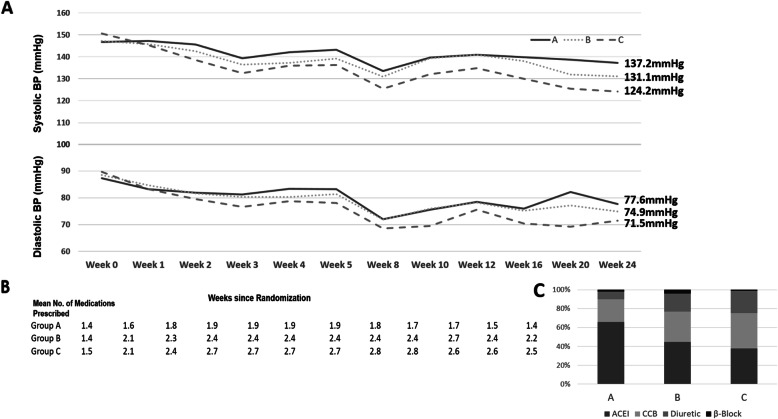
Mean systolic blood pressure (BP) of three treatment groups during the study visits. Panel **a**: mean systolic blood pressure (SBP) during the treatment period in the standard-BP-control group, the moderate-BP-control group, and the intensive-BP-control group was 137.2 mmHg, 131.1 mmHg, and 124.2 mmHg, respectively, while the corresponding DBP was 77.6 mmHg, 74.9 mmHg. and 71.5 mmHg in each of the three groups, respectively, by the end of 6 months of follow-up. Panel **b**: the mean number of antihypertensive drugs prescribed at enrollment was 1.4, 1.4, and 1.5 among the standard-BP-control group, the moderate-BP-control group, and the intensive-BP-control group, respectively. After 6 months of follow-up, the mean number of drugs prescribed was 1.4, 2.2, and 2.5, per group, respectively. Panel C: the distribution of antihypertensive drugs used in the different groups

The mean SBP at the end of the 6-month visit in the standard-BP-control group (A), the moderately intensive-BP-control group (B), and the intensive-BP-control group (C) was 137.2 mmHg, 131.1 mmHg, and 124.2 mmHg, respectively, while the corresponding DBPs were 77.6 mmHg, 74.9 mmHg, and 71.5 mmHg, for each group, respectively (Fig. 2A).

The mean number of antihypertensive drugs prescribed at baseline enrollment were 1.4, 1.4, and 1.5 among the standard-BP-control group (A), the moderately intensive-BP-control group (B), and the intensive-BP-control group (C), respectively, and, at the end of 6-month visit, were 1.4, 2.2, and 2.5, respectively (Fig. 2B). The distribution of antihypertensive drugs used in the different groups was shown in Fig. 2C.

Decreased SBP and DBP was expressed as ΔSBP and ΔDBP (which equals to SBP at week “x” – SBP at week “0”), respectively. After 6 months of antihypertensive treatment, the absolute decrease in SBP in Groups A, B, and C was 9.5 mmHg, 16.1 mmHg, and 26.4 mmHg, respectively, while the absolute decrease in DBP was 9.7 mmHg, 13.6 mmHg, and 18.2 mmHg, respectively (Table 2).

**Table 2:** Decrease in systolic blood pressure (ΔSBP) and decrease in diastolic blood pressure (ΔDBP) at each follow-up visit

	Week 1	Week 2	Week 3	Week 4	Week 5	Week 8	Week 10	Week 12	Week 16	Week 20	Week 24
**ΔSBP**^**a**^											
A^b^	0.5	− 1.1	− 7.4	− 4.7	− 3.5	− 13.2	− 7	− 5.7	− 6.8	− 8	− 9.5
B	− 1.4	− 4.6	− 10.8	− 9.9	− 8	− 16.3	− 7.9	− 6.3	− 9.2	− 15.3	− 16.1
C	− 5.3	− 12.1	− 18.1	− 14.6	− 14.4	− 25.1	− 18.5	− 15.8	− 20.6	− 25.2	− 26.4
**ΔDBP**											
A	− 4.1	− 5.4	− 6.1	− 3.9	− 4.1	− 15.3	− 11.7	− 8.9	− 11.4	− 5.2	− 9.7
B	− 3.8	− 6.9	− 8.2	− 8.2	− 7.2	− 16.7	− 12.6	− 10.3	− 13.3	− 11.4	− 13.6
C	− 6.5	− 10.2	− 13.1	− 11	− 11.7	− 21.2	− 20.2	− 14.2	− 19.3	− 20.5	− 18.2

^a^*ΔSBP* SBP (week “x”) – SBP (week 0); *ΔDBP* DBP (week “x”) – DBP (week 0)

^b^*A* standard group, *B* moderately intensive group, *C* intensive group

After 6 months of treatment, for the standard-BP-control group (A), 83% achieved SBP < 150 mmHg (29% of participants had a mean SBP in the BP target window of 140–150 mmHg, but 14% were in the 130–140 mmHg window and 40% were in the < 130 mmHg group). For the moderately intensive-BP-control group (B), 80% achieved SBP < 140 (37% of participants had a mean SBP in the target window of 130–140 mmHg, but 43% were in the < 130 mmHg group). For the intensive-BP-control group (C), 73% of participants had a mean SBP < 130 mmHg (6% of participants had a mean SBP in the target window of 140–150 mmHg, and 18% were in the target window of 130–140 mmHg) (see Supplemental Fig. 2)

### HBPM and CASP

In this study, 98 participants (93%) agreed to self-monitor their BP using an electronic sphygmomanometer for HBPM; and 94 of the 98 participants completed the HBPM according to protocol, including 29 men (30.9%) with a mean age of 71.0 (± 5.2) years and 65 women (69.1%) with a mean age of 67.2 (± 5.0) years. There was a consistent trend between office visit BP and HBPM (2 h after taking medication) among the three BP-control groups at each titration period (Supplemental Figure 1A). CASP was also measured at weeks 6, 12, 24. Consistent trends were also observed between CASP and office visit BP among the three groups at each titration period (Supplemental Figure 1B). CASP was generally lower than that of the office visit BP (Supplemental Table 2). At the end of 6-month titration, the difference in SBP between CASP and office visit BP was − 7.1 ± 8.0 in the standard-BP-control group (A), − 9.6 ± 8.2 in the moderately intensive-BP-control group (B), and − 9.0 ± 9.0 in the intensive-BP-control group (C).

### Adverse events (AEs)

There were no severe AEs recorded and no direct or close relationships between the occurrence of an AE and the BP titration. As shown in Table 3, there were no significant differences in AE occurrence, especially hypotension, between the intensive-BP-control group and the other groups.

**Table 3:** Adverse events among the three blood pressure (BP) control groups

Adverse event (Number of patients rate (%))	Standard BP control	Moderate BP control	Intensive BP control	Total
Cold symptoms	7 (21%)	9 (26%)	7 (20%)	23
Dry cough	5 (15%)	5 (14%)	3 (9%)	13
Vertigo	3 (9%)	3 (9%)	3 (9%)	9
Arthralgia	0	1 (3%)	2 (6%)	3
Epigastric pain	1 (3%)	1 (3%)	0	2
Palpitations	0	0	1 (3%)	1
Drug allergy	1 (3%)	0	0	1
Skin disease	0	1 (3%)	0	1
Blurred vision	0	0	1 (3%)	1
Hypotension	0	0	0	0
**Total**	17 (49%)	20 (57%)	17 (49%)	53

## Discussion

To our knowledge, this is the first randomized trial to test the feasibility and safety of the BP-control protocol (including medication titration) to achieve three different BP-control targets in rural, Chinese hypertensive patients. This trial gained real-world experience and laid the foundation for a future large-scale BP target study. Below we discuss what we learned from this trial and how it relates to the literature.

### BP-control targets

Given the lack of consensus on optimal BP targets in the Chinese population, we chose three SBP targets based on American Heart Association previous and new BP guidelines [2, 11, 16] and findings from the two relevant trials: SPRINT and ACCORD [10, 17]. Our goal was to evaluate how likely each of the BP targets can be safely achieved in rural, Chinese hypertensive patients, a population with a low BP-control rate and at high-risk of stroke. Our ultimate goal of the management of hypertension is for the prevention of end-organ damage, including stroke, cardiovascular events, and renal dysfunction.

The benefits of BP-lowering were demonstrated in RCTs of hypertensive patients. The following trials contributed to the changes in the BP targets in the major hypertension management guidelines from 2000 to 2018: the Hypertension in the Very Elderly Trial (HYVET 2003) [18]; the Action to Control Cardiovascular Risk in Diabetes trial (ACCORD 2010) [17]; the Valsartan in Elderly Isolated Systolic Hypertension study (VALISH 2010) [19]; the Secondary Prevention of Small Subcortical Strokes trial (SPS3 2013) [20]; the Systolic Blood Pressure Intervention trial (SPRINT 2015) [10]; and the Heart Outcome Prevention Evaluation-3 trial (HOPE-32016) [21].

Of note, in contrast to the findings of “SPRINT,” which showed a benefit of tighter BP control, the ACCORD trial showed no significant difference in cardiovascular events and all-cause mortality between the intensive treatment (mean SBP 119.3 mmHg) and the standard treatment (mean SBP 133.5 mmHg); cardiovascular events and death from cardiovascular causes (hazard ratio (HR) 0.88, 95% CI 0.73–1.06, *p* = 0.20). However, the cardiovascular events observed in the ACCORD trial were mainly related to ischemic heart disease, but the prevalence of cerebrovascular disease was significantly reduced in the intensive-treatment group (HR 0.59, 95% CI 0.39–0.89, *p* = 0.01). There were important differences between the two trials. The ACCORD trial enrolled participants with diabetes exclusively, whereas SPRINT excluded participants with diabetes; in addition, the sample size differed (4733 in ACCORD vs. 9361 in SPRINT). The ACCORD trial also used a factorial design that included comparisons of standard and intensive glycemic and lipid treatment targets in the same trial. SPRINT enrolled an older cohort (mean age, 68 years vs. 62 years in the ACCORD trial), with 28% of the participants being 75 years of age or older, and included participants with chronic kidney disease.

Limited data were available for Asian populations. A recent study among 248,8101 Koreans aged 20 through 39 years found that stage 1 hypertension (SBP 130–139 mmHg or DBP 80–89 mmHg) was associated with an increased risk of subsequent cardiovascular disease (HR 1.25 for men; 1.27 for women) during a median follow-up duration of 10 years. Among Koreans, young adults with hypertension, defined by the 2017 ACC/AHA criteria, may be at increased risk of cardiovascular disease [22].

### Choice of antihypertensive drugs

Drug choice is related to the clinical indications, cost, availability, insurance coverage, and patient preference. In the ACCORD trial, a strategy of treatment to specific SBP goals was tested, rather than testing any specific drug regimen. All major classes of antihypertensive drugs and many combination medications were provided by the study. All antihypertensive regimens were to include a drug class that had demonstrated efficacy in reducing cardiovascular events in participants with diabetes: diuretics, β-blockers, CCBs, ACEIs, or angiotensin-II- receptor blockers (ARBs). The treatment algorithms of SPRINT were similar to the ACCORD trial. The SPRINT investigators also prescribed other antihypertensive medications (not provided by the study). The protocol encouraged, but did not mandate, the use of drug classes with the strongest evidence for reduction in cardiovascular outcomes, including thiazide-type diuretics (encouraged as the first-line agent), loop diuretics (for participants with advanced chronic kidney disease), and β-adrenergic blockers (for those with coronary artery disease). Chlorthalidone was encouraged as the primary thiazide-type diuretic, and amlodipine as the preferred CCB. In the International Verapamil-Trandolapril Study (INVEST) [23], patients were randomly assigned to either a calcium antagonist (verapamil sustained release) vs. a non-calcium antagonist (atenolol). Trandolapril and/or hydrochlorothiazide was administered to achieve BP goals.

In this present trial, the antihypertensive drugs were provided at no cost to the participants; and all the antihypertensive regimens included drug classes that had been shown to result in a reduction in stroke or cardiovascular events [24]. For all participants, the initial therapy was a daily oral dose of one tablet of enalapril-folic acid (containing 10 mg of enalapril and 0.8 mg of folic acid) because the Hcy level of all participants was > 10 μmol/L [25]. The next step used amlodipine or hydrochlorothiazide. We also allowed β-blockers to achieve the SBP target.

### Feasibility

Feasibility encompasses the likelihood of lowering BP to the prespecified target, the number of drugs required to achieve that goal, and whether patients can tolerate and comply with the regimen. In this trial, all patients completed the 6-month follow-up with the exception of only one participant in the intensive-BP-control group (the patient had to travel out of town for an emergency). After 6 months of BP medication titration, 83%, 80%, and 73% of the patients attained a BP level below the specified SBP target for Groups A, B, C, respectively. At baseline enrollment, the mean number of antihypertensive drugs prescribed was 1.4, 1.4, and 1.5 among the three groups. After 6 months of follow-up, the number of drugs prescribed were 1.4, 2.2, and 2.5 (Fig. 2B). In SPRINT the mean number of BP medications was 2.8 in the intensive treatment group and 1.8 in the standard treatment group. In the ACCORD study the mean number of medications after the first year was 3.4 (95% CI 3.4–3.5) in the intensive-therapy group and 2.1 (95% CI 2.1–2.2) in the standard-therapy group.

In the process of BP medication titration, SBP fluctuated somewhat as in week 8, and we considered that ambient temperature was a potential contributor [26]. As part of BP measurements, we also recorded the ambient temperature. As shown in Supplemental Fig. 3, ambient temperature was correlated with BP levels. On the other hand, between the week-5 visit and week-8 visit, there is a Spring Festival Holiday with sufficient rest time, which is also good for BP control.

### Safety

Safety issues were related to side effects of the specific antihypertensive drug used; safety surrounding the use of multiple drugs in combination; and safety related to the BP target and the corresponding risk of hypotension. The major side effects observed in the current study were cold symptoms, dry cough, and vertigo, all of which were similar between the three groups (Table 3). No SAEs were recorded. The BP-control protocols were overall safe without any major AE for all three target groups.

### Modality of BP monitoring

The current study tested different modalities of BP measurements: office visits, self-monitored HBPM, and CASP and examined their relationships. We found a consistent pattern of BP control between HBPM and office-visit BP measurements. In addition, there was a general 8–9 mmHg difference between CASP and office visit BP.

## Strengths and limitations of this study

This pilot randomized trial was the first step to address critical questions: what is the optimal BP-control target and how to achieve it in the Chinese population? This trial aimed to evaluate the feasibility and safety of achieving prespecified BP targets (< 150, < 140, and < 130 mmHg) using a standard BP-control protocol among hypertensive patients in rural China, which constitutes over 61.2% of Chinese population. This trial fully considered the rural Chinese population’s characteristics such as socio-economic status, compliance, education level, and lifestyle, which are quite different from western populations. This trial has the following limitations: the sample size was small. The study had a short duration and was unable to evaluate long-term health outcomes. It was conducted in rural, Chinese hypertensive patients, so generalization of the trial findings to other population requires caution. There are more women ‘left behind’ than men in rural China, so we have included more women. Salt intake is high in northern China, but apart from lifestyle modification, we have not accurately measured salt intake in this study.

## Conclusion

The findings from this pilot trial suggest that all three BP targets (< 150, < 140, and < 130 mmHg) can be safely achieved in hypertensive patients in rural China, without a history of stroke and cardiovascular events, using our BP-control medication titration protocol. The next step would be to determine the long-term effects of different BP targets on end-organ diseases, which would require both a large and longer-term trial.

## Trial status

This pilot trial presented above has been completed.

## Supplementary information

**Additional file 1: Supplemental Figure 1.** Comparison of office visit systolic blood pressure (SBP) with home blood pressure measurement (HBPM) and central aortic systolic pressure (CASP). Panel A: 94 participants completed the HBPM according to the protocol. There was a consistent trend between office visit BP and HBPM (2 h after taking medication) among the standard BP-control group, the moderate-BP control group, and the intensive-BP-control group at each titration period. Panel B: CASP was also measured at weeks 6, 12, and 24. There was a consistent trend between CASP and office visit BP among the standard-BP-control group, the moderate-BP-control group, and the intensive-BP-control group at each titration period.

**Additional file 2: Supplemental Figure 2.** Mean systolic blood pressure (SBP) in the target window of three treatment groups at each visit. After 6 months of titration, for the standard-BP-control group, 29% of participants had a mean SBP in the target window of 140–150 mmHg, 14% were in the 130–140 mmHg window and 40% were in the < 130 mmHg group; for the moderate-BP-control group, 37% of participants had a mean SBP in the target window of 130–140 mmHg, and 43% were in the < 130 mmHg group; for the intensive-BP-control group, 73% of participants had a mean SBP in the < 130 mmHg group.

**Additional file 3: Supplemental Figure 3.** Systolic blood pressure (SBP) fluctuated in the process of BP titration and ambient temperature recorded. In the process of BP medication titration, SBP did not always decrease, but fluctuated in the middle. Ambient temperature affected BP control.

**Additional file 4: Supplemental Table 1.** Classes of antihypertensive agents.

## Abbreviations

SBP: Systolic blood pressure
DBP: Diastolic blood pressure
SPRINT: Systolic Blood Pressure Intervention Trial
HHcy: Hyperhomocysteinemia
HBPM: Home blood pressure measurement
CASP: Central aortic systolic pressure
FDC: Fixed-dose combination
SAEs: Serious adverse events
