# Supplementary figures and images for: Achieving blood pressure control targets in hypertensive patients of rural China – a pilot randomized trial
Source: Trials. 2020 Jun 11;21:515. doi: 10.1186/s13063-020-04368-1 (PMC7291427; doi:10.1186/s13063-020-04368-1)

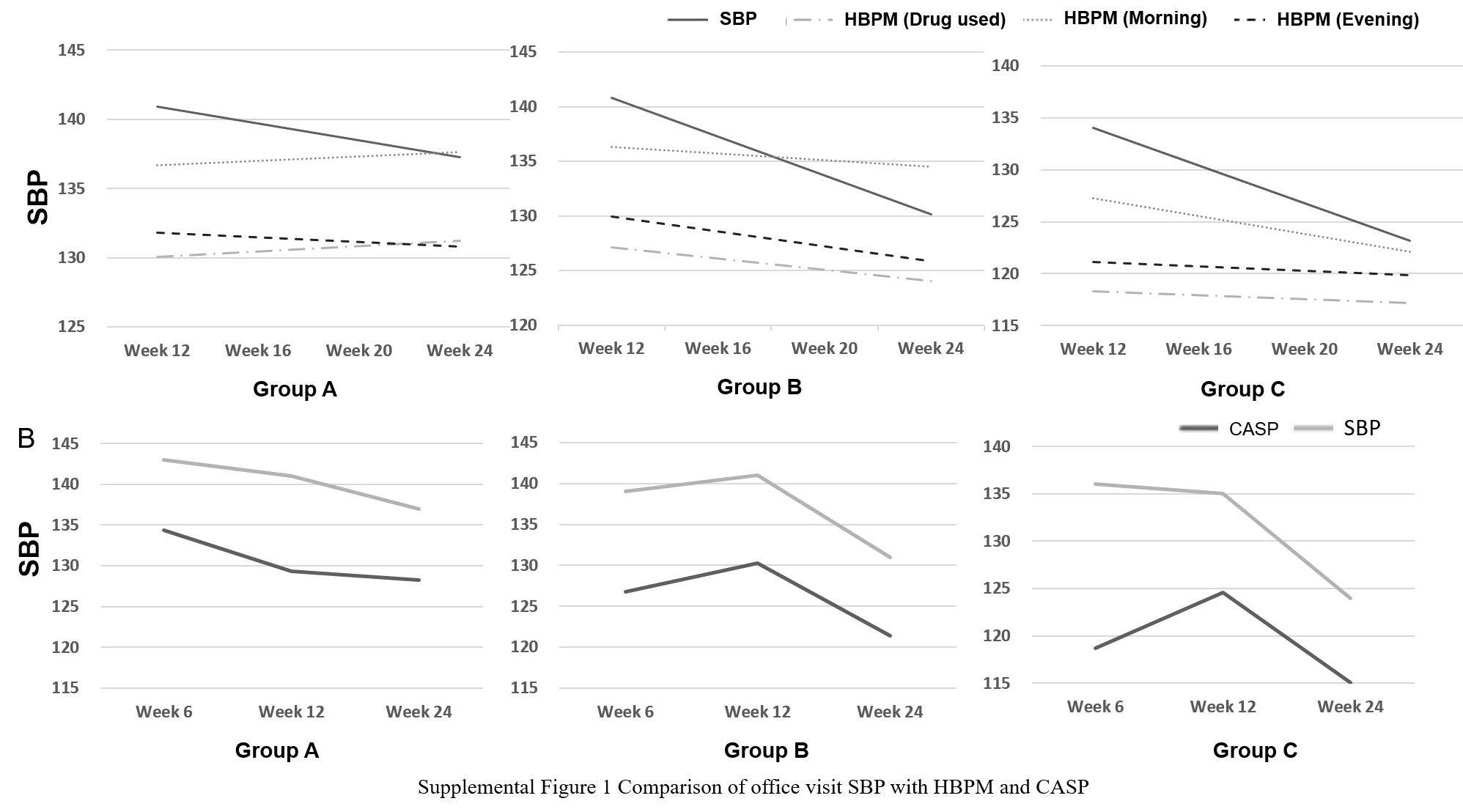

Supplement: Supplementary file 1 — Additional file 1: Supplemental Figure 1. Comparison of office visit systolic blood pressure (SBP) with home blood pressure measurement (HBPM) and central aortic systolic pressure (CASP). Panel A: 94 participants completed the HBPM according to the protocol. There was a consistent trend between office visit BP and HBPM (2 h after taking medication) among the standard BP-control group, the moderate-BP control group, and the intensive-BP-control group at each titration period. Panel B: CASP was also measured at weeks 6, 12, and 24. There was a consistent trend between CASP and office visit BP among the standard-BP-control group, the moderate-BP-control group, and the intensive-BP-control group at each titration period. [file 13063_2020_4368_MOESM1_ESM.tif]

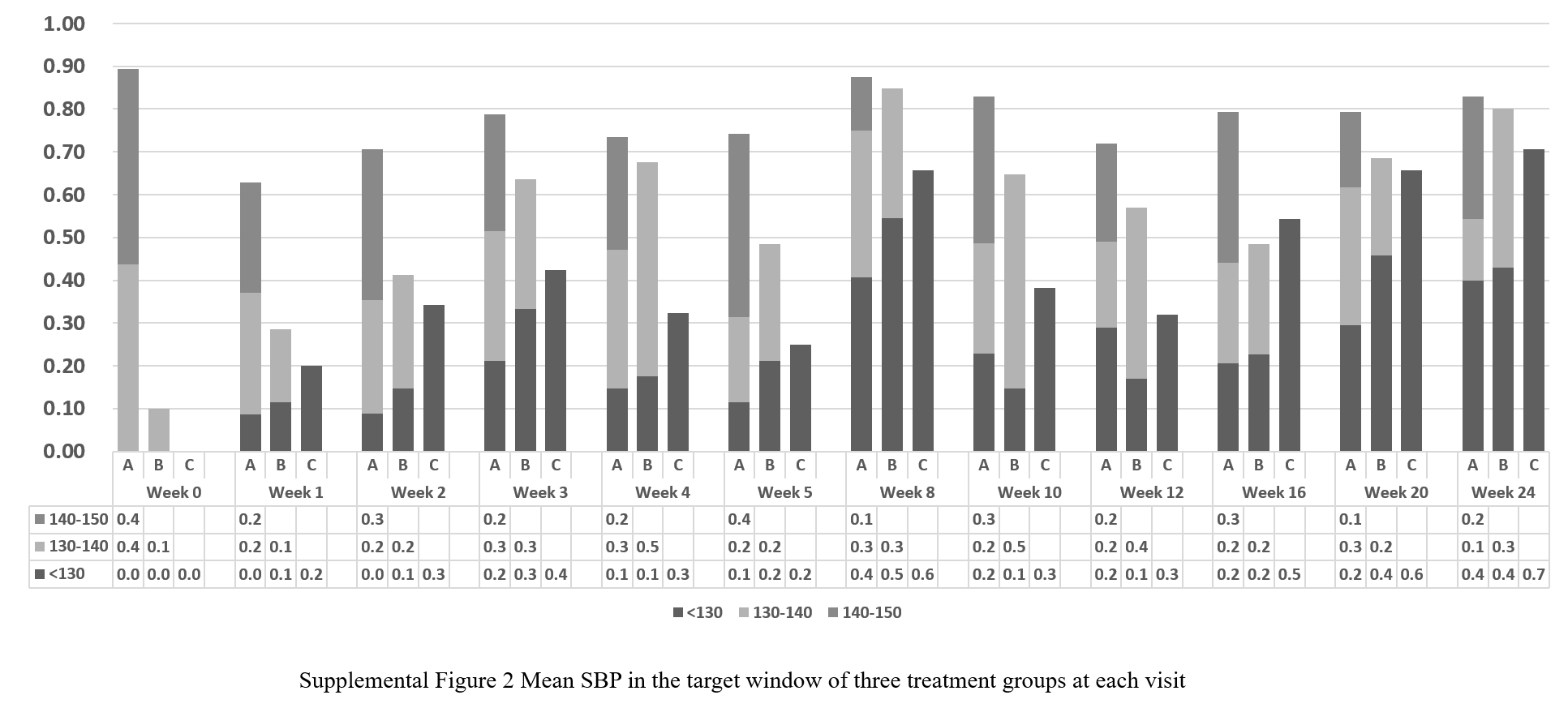

Supplement: Supplementary file 2 — Additional file 2: Supplemental Figure 2. Mean systolic blood pressure (SBP) in the target window of three treatment groups at each visit. After 6 months of titration, for the standard-BP-control group, 29% of participants had a mean SBP in the target window of 140–150 mmHg, 14% were in the 130–140 mmHg window and 40% were in the < 130 mmHg group; for the moderate-BP-control group, 37% of participants had a mean SBP in the target window of 130–140 mmHg, and 43% were in the < 130 mmHg group; for the intensive-BP-control group, 73% of participants had a mean SBP in the < 130 mmHg group. [file 13063_2020_4368_MOESM2_ESM.tif]

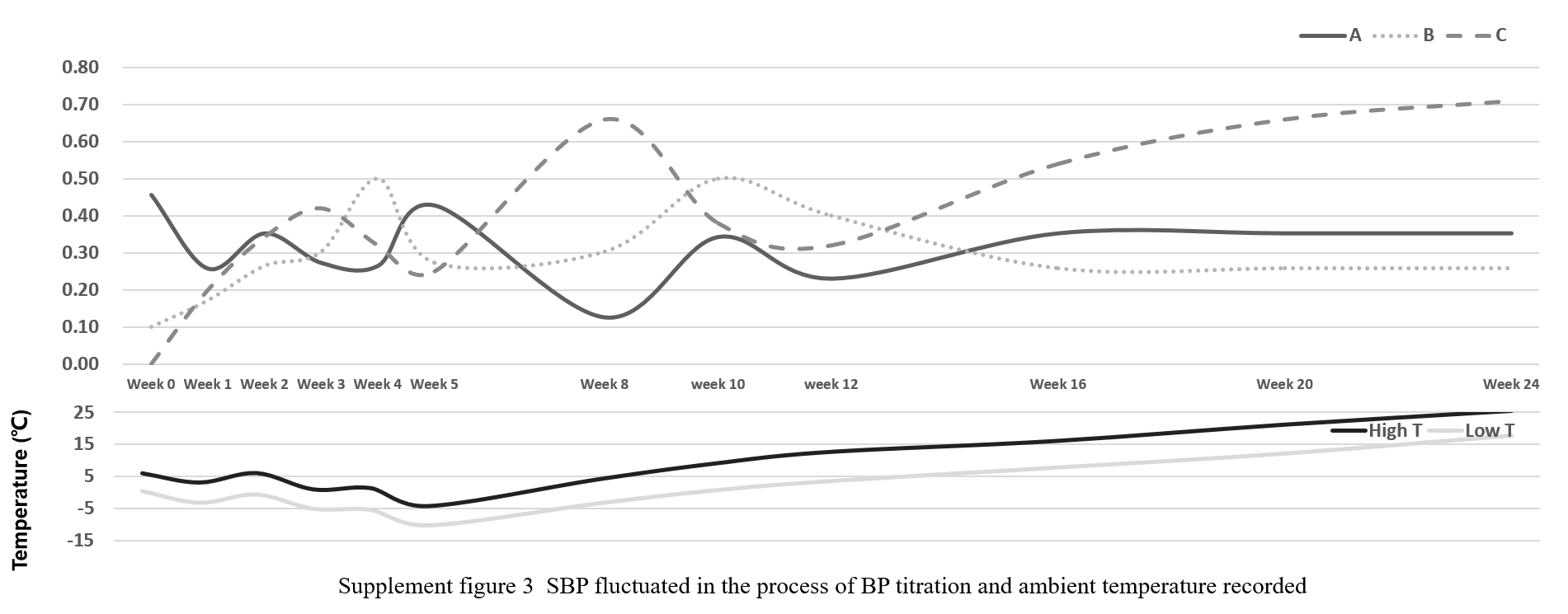

Supplement: Supplementary file 3 — Additional file 3: Supplemental Figure 3. Systolic blood pressure (SBP) fluctuated in the process of BP titration and ambient temperature recorded. In the process of BP medication titration, SBP did not always decrease, but fluctuated in the middle. Ambient temperature affected BP control. [file 13063_2020_4368_MOESM3_ESM.tif]
