# Supplementary material for: Achieving blood pressure control targets in hypertensive patients of rural China – a pilot randomized trial
Source: Trials. 2020 Jun 11;21:515. doi: 10.1186/s13063-020-04368-1 (PMC7291427; doi:10.1186/s13063-020-04368-1)
Supplement: Supplementary file 4 — Additional file 4: Supplemental Table 1. Classes of antihypertensive agents. [file 13063_2020_4368_MOESM4_ESM.pdf]

Supplemental Table 1 Classes of antihypertensive agents

Step	Type of antihypertension drug	Antihypertension drug	Available strengths	Usual dose range per day	Frequency
1	Ace inhibitor (ACEI)	Enalapril+Folic acid	10mg+0.8mg	5.4mg-10.8mg	1-2
2*	Diuretic	Hydrochlorothiazide	12.5mg,25mg	12.5-25mg	1
3*	Calcium channel blocker (CCB)	Amlodipine	5mg,10mg	2.5-10mg	1-2
4	Beta blocker (β-B)	Metoprolol	25mg,47.5mg	12.5-47.5mg	1-2

*2 or 3 were randomly chosen.

Supplemental Table 2

Group	Office Visit SBP	CASP	ΔSBP[#]
A	137.2 ± 14.9	129.1 ± 13.7	-7.1 ± 8.0
B	131.1 ± 14.0	121.4 ± 15.3	-9.6 ± 8.2
C	124.2 ± 13.7	115.1 ±13.8	-9.0 ± 9.0

[#]ΔSBP = CASP-Office Visit SBP

A =Standard Group B=Moderately Intensive Group C= Intensive Group

Supplemental Table 3

Group	Current study		Group	SPRINT	Group	ACCORD
	SBP(Mean) mmHg	SBP(6 months) mmHg		SBP(Mean) mmHg		SBP(Mean) mmHg
A >140, <=150	141.3	137.2	A<=140	134.6	A<=140	133.5
B >130, <=140	138.4	131.1	B<=120	121.5	B<=120	119.3
C <=130	134.4	124.2				
